# Anti-interferon-γ autoantibody-mediated immunodeficiency with disseminated *Mycobacterium massiliense* and secondary Sweet syndrome: A case report

**DOI:** 10.1097/MD.0000000000049079

**Published:** 2026-05-29

**Authors:** Jiajia Chen, Zihao Han, Jiajun Liu, Lili Wang, Jiguang Guo

**Affiliations:** a Department of Respiratory and Critical Care Medicine, The People’s Hospital of Rongchang District, ChongQing, China; b Department of Ophthalmology, Chongqing General Hospital, ChongQing, China; c Department of Pathology, The People’s Hospital of Rongchang District, ChongQing, China; d Department of Nephrology, The People’s Hospital of Rongchang District, ChongQing, China.

**Keywords:** adult-onset immunodeficiency, anti-interferon-gamma autoantibodies, disseminated non-tuberculous mycobacterial disease, *Mycobacterium massiliense*, Sweet syndrome

## Abstract

**Rationale:**

The co-occurrence of adult-onset immunodeficiency syndrome mediated by anti-interferon-γ autoantibodies (AIGA-AOID), disseminated non-tuberculous mycobacterial (NTM) infection, and Sweet syndrome is a recognized but underreported triad, particularly in East Asian populations. Detailed case reports emphasizing diagnostic approach and integrated management remain valuable for improving clinical recognition.

**Patient concerns:**

A 70-year-old Asian female presented with a 1-year history of a progressive neck mass, a 6-month history of recurrent fever, and a painful cutaneous eruption on the face, trunk, and limbs.

**Diagnoses:**

Evaluations revealed disseminated *Mycobacterium massiliense* infection involving lymph nodes, lung, and skin. Skin biopsy was consistent with Sweet syndrome. High-titer anti-interferon-γ autoantibodies (1:2500) were detected, confirming AIGA-AOID in the absence of HIV or other immunosuppression.

**Interventions:**

Combined therapy was initiated: rituximab (two weekly doses) with corticosteroids, and a 12-month oral antimycobacterial regimen (clarithromycin, moxifloxacin, linezolid).

**Outcomes:**

The patient showed marked clinical improvement within 2 weeks, with resolution of fever and skin healing. Follow-up imaging at 3 months demonstrated significant regression of lymphadenopathy and lung consolidation.

**Lessons:**

Sweet syndrome can be a presenting sign of disseminated NTM disease. In immunocompetent-appearing adults, especially in endemic regions, this should prompt investigation for AIGA-mediated immunodeficiency. Early diagnosis and combined immunomodulatory-antimicrobial therapy are crucial for successful outcomes.

## 1. Introduction

The emergence of adult-onset immunodeficiency syndrome associated with neutralizing anti-interferon-γ autoantibodies represents a distinct clinical entity, predominantly reported in East and Southeast Asian populations.^[1]^ This syndrome predisposes otherwise immunocompetent-appearing adults to severe, disseminated infections with intracellular pathogens, most notably non-tuberculous mycobacteria (NTM).^[2]^ Disseminated NTM disease, a classic opportunistic infection in this setting, often presents a diagnostic challenge due to its nonspecific systemic and focal symptoms.

Sweet syndrome (acute febrile neutrophilic dermatosis) is a reactive inflammatory condition that can be idiopathic, paraneoplastic, or triggered by infections.^[3]^ Notably, an increasing number of reports have linked Sweet syndrome to disseminated mycobacterial infections, particularly in the context of underlying immunodeficiencies.^[4]^ While the triad of AIGA-mediated immunodeficiency, disseminated NTM, and Sweet syndrome has been described, detailed case reports illustrating the diagnostic pathway and integrated management remain valuable for raising clinical awareness. To the best of our knowledge, this is the first detailed report of AIGA-AOID with disseminated M. massiliense manifesting as Sweet syndrome, highlighting an integrated diagnostic approach using metagenomic next-generation sequencing and a successful dual immunomodulatory-antimicrobial strategy. We herein present a case that exemplifies this complex interplay.

## 2. Case presentation

Ethical approval for this study was obtained from the Ethics Committee of The People Hospital of Rongchang District, and written informed consent was provided by the patient for publication of this case report and any accompanying images.

**Figure 1.**
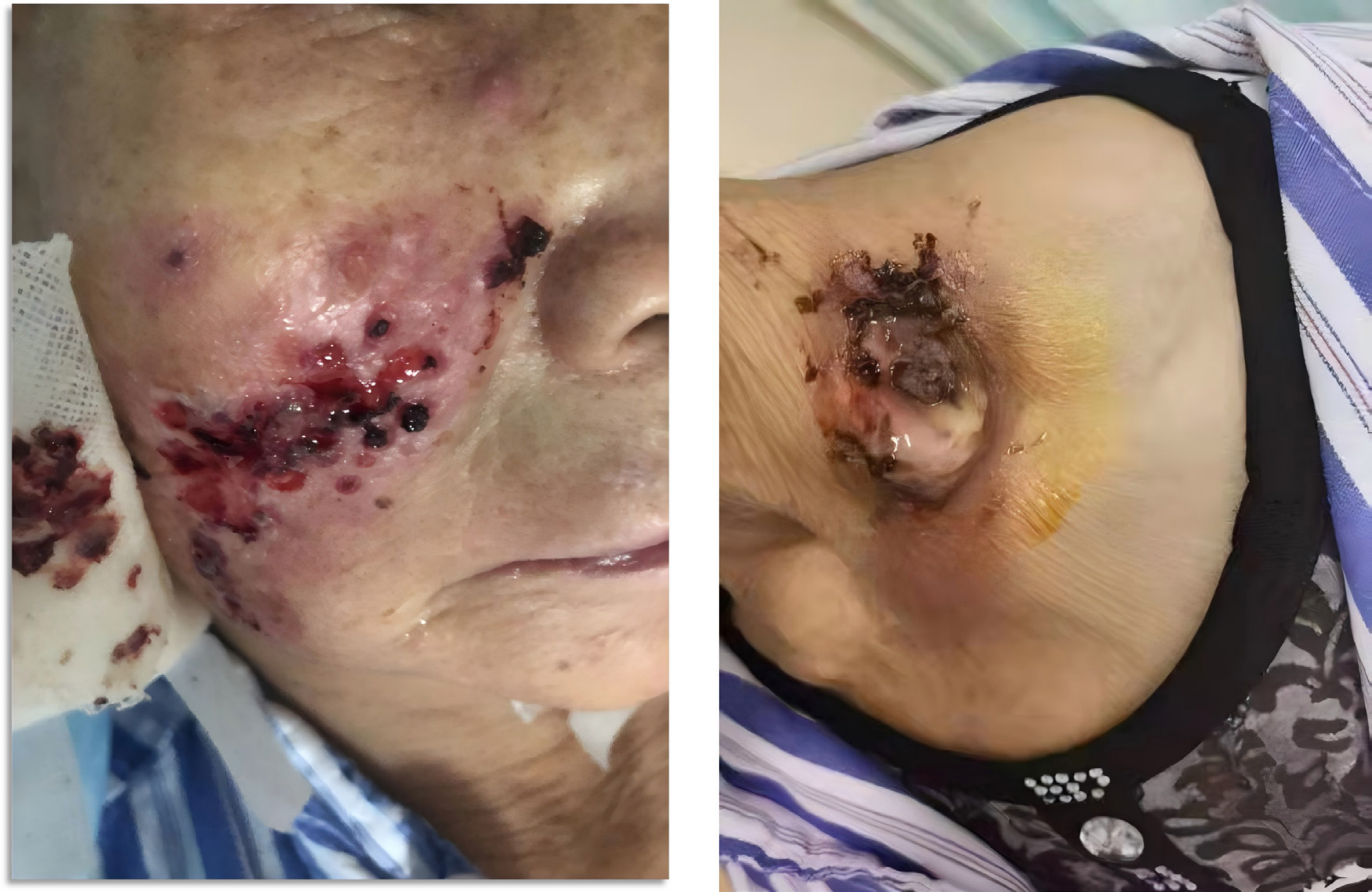
Painful erythematous rash on the face and cutaneous erosion with crusting on the left side of the neck.

A 70-year-old Asian female was admitted to our hospital in December 2024, presenting with a 1-year history of a progressively enlarging, painless mass in the left side of her neck and a 6-month history of recurrent fever and generalized skin rash. Over the 6 months preceding admission, she had developed intermittent fever (up to 38.5°C), fatigue, and a nonproductive cough. Concurrently, a painful erythematous rash with vesicles and pustules appeared on her face (Fig. 1), trunk, and limbs, with the facial lesions progressing to erosion and crust formation. She received empiric antibiotic therapy with piperacillin-tazobactam for 5 days after admission, which yielded no clinical improvement.

Physical examination on admission revealed a febrile patient (temperature 39.3°C) with extensive, tender, eroded, and crusted plaques on the face, ears, and left side of the neck. Multiple enlarged, soft, non-tender lymph nodes were palpable in the left supraclavicular fossa, the largest measuring 2.5 cm in diameter. Cardiopulmonary and abdominal examinations were unremarkable. Laboratory investigations revealed leukocytosis (12.95 × 10^9^/L) with neutrophilia (80.3%), and elevated inflammatory markers (C-reactive protein 119.13 mg/L, erythrocyte sedimentation rate 89 mm/h). Procalcitonin was within the normal range. Tests for tuberculosis (PPD skin test, T-SPOT.TB), sputum smear for acid-fast bacilli, blood cultures, and (1,3)-β-D-glucan/galactomannan assays were negative. Liver and kidney function tests were normal. Serology for hepatitis B surface antibody, hepatitis C virus antibody, syphilis, and HIV was nonreactive. Autoimmune serologies, including antinuclear antibody, antineutrophil cytoplasmic antibody, and an autoantibody profile, were negative, as were panels for tumor markers.

**Figure 2.**
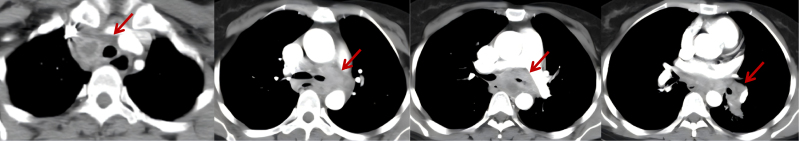
Contrast-enhanced chest CT findings, with red arrows indicating multiple enlarged lymph nodes.

**Figure 3.**
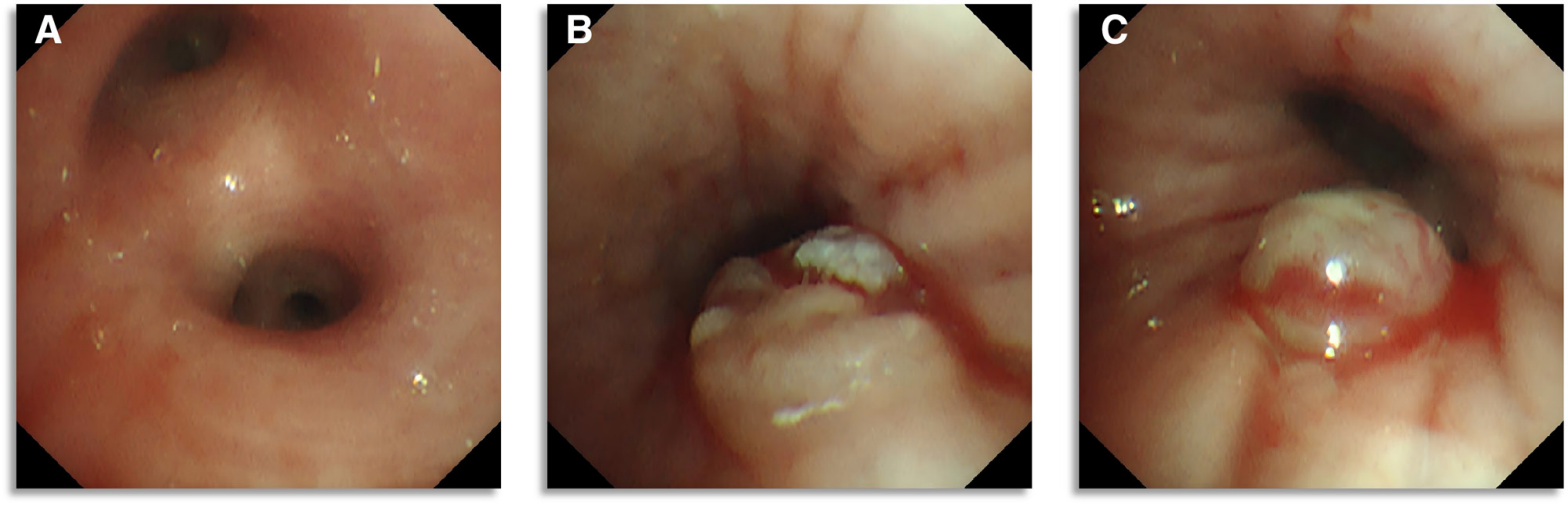
Bronchoscopy findings. (A) Opening of the left upper lobe; (B) Opening of the dorsal segment of the left lower lobe; (C) Opening of the basal segments of the left lower lobe.

At this stage, the main differential diagnoses included lymphoma, disseminated tuberculosis, deep fungal infection, and systemic autoimmune disease. The presence of extensive lymphadenopathy and negative initial microbiologic and serologic workup prompted tissue sampling. Imaging studies included a contrast-enhanced chest CT (Fig. 2), which demonstrated bronchial wall thickening and luminal narrowing in the apical segment of the left lower lobe, surrounded by streaky opacities showing heterogeneous enhancement. A linear density was observed within the lumen of the left main bronchus. Significant lymphadenopathy was noted in the mediastinum, left pulmonary hilum, and left axilla. Neck ultrasonography identified multiple partially fused hypoechoic nodules in the left supraclavicular fossa (largest 30.0 × 20.6 mm), which lacked a visible hilum and showed no significant vascularity on color Doppler imaging. Bronchoscopy revealed an endobronchial mass in the left lower lobe bronchus, featuring an irregular surface covered with focal white necrotic material (Fig. 3). Endobronchial ultrasound-guided transbronchial needle aspiration (EBUS-TBNA) confirmed enlarged lymph nodes at stations 4L, 7, and 4R.

**Figure 4.**
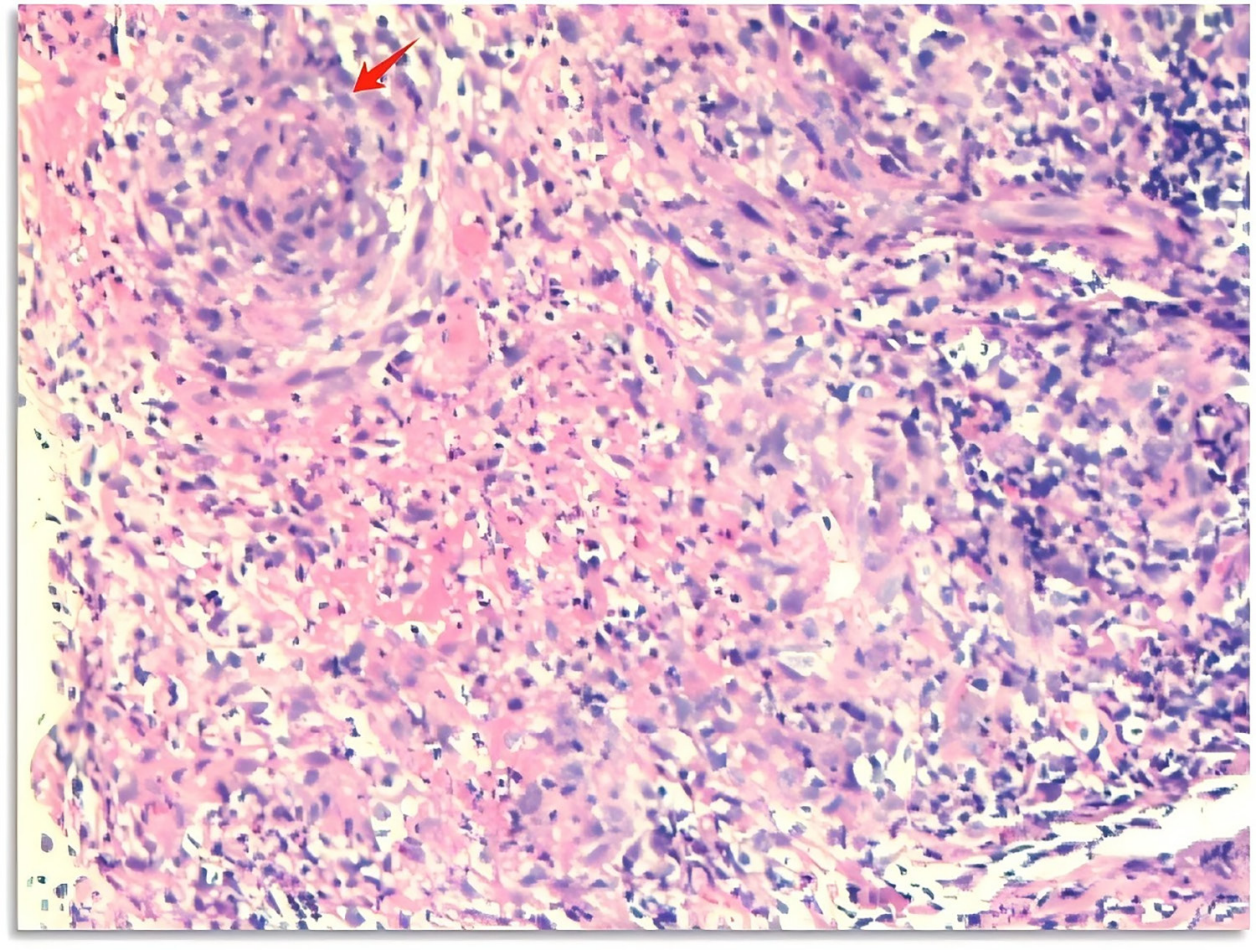
Supraclavicular lymph node biopsy findings, with red arrows indicating granulomatous inflammation.

Histopathological examination of a skin biopsy showed marked papillary dermal edema and a dense, diffuse infiltrate composed predominantly of mature neutrophils, without evidence of leukocytoclastic vasculitis. Special stains for microorganisms (acid-fast and PAS) were negative. These findings fulfilled the major criteria for Sweet syndrome (acute onset of painful erythematous plaques and classic histopathology) together with minor criteria (fever > 38°C, elevated ESR/CRP, and subsequent rapid response to corticosteroids), effectively excluding direct cutaneous mycobacterial infection. A biopsy of a supraclavicular lymph node revealed granulomatous inflammation with necrosis (Fig. 4). A biopsy from the station 7 lymph node also showed granulomatous inflammation and was subsequently positive for acid-fast staining. Metagenomic next-generation sequencing (mNGS) of an aspirate from the station 7 lymph node identified *Mycobacterium massiliense* (377 sequence reads). Due to the limited volume of the aspirate, mycobacterial culture could not be performed. However, the mNGS finding was considered valid because of the high, specific read count, the concordant presence of acid-fast bacilli on tissue staining, and the rigorous negative controls employed. No additional PCR confirmation or phenotypic susceptibility testing was available. Given the findings of disseminated NTM infection in an HIV-negative patient without other known causes of immunodeficiency, an underlying immune disorder was suspected. The decision was therefore made to test for anti-IFN-γ autoantibodies (AIGA). Serological testing confirmed high-titer AIGA at 1:2500, using a commercial ELISA kit with a positive cutoff titer of 1:100. Although a neutralizing activity assay was not performed, such elevated titers are invariably associated with functional neutralization and are diagnostic for AIGA-mediated immunodeficiency in the appropriate context.

Based on these findings, the final diagnoses were: adult-onset immunodeficiency syndrome mediated by AIGA; disseminated M. massiliense infection involving lymph nodes, lung, and skin; and Sweet syndrome secondary to the mycobacterial infection. The treatment regimen combined immunomodulation and antimicrobial therapy. The patient received rituximab (375 mg/m^2^, administered weekly for 2 doses) along with intravenous methylprednisolone (1.0 mg/kg/day, with a weekly taper of 10% initiated after 2 weeks). Concurrently, a 12-month oral antimycobacterial regimen was started, consisting of clarithromycin (500 mg twice daily), moxifloxacin (400 mg once daily), and linezolid (600 mg twice daily).

**Figure 5.**
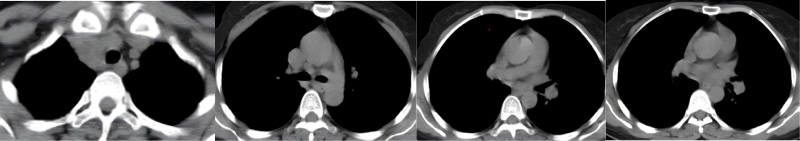
Non-contrast chest CT scan at 3 months follow-up.

The patient clinical condition improved markedly within 2 weeks of initiating the combined immunomodulatory and antimicrobial therapy. Her fever resolved, and the skin lesions began to heal. A follow-up chest CT scan obtained 3 months later demonstrated significant regression of the mediastinal lymphadenopathy and lung consolidation (Fig. 5). The patient remains on a sustained regimen of antimycobacterial therapy and is being monitored for potential rituximab re-dosing, guided by her clinical status and CD19 + B-cell counts. Unfortunately, due to relocation to another province, the patient was lost to further follow-up at our center after the 3-month visit, precluding collection of long-term outcome data.

## 3. Discussion and conclusion

This case provides a compelling illustration of the intricate interplay between a specific immunodeficiency, a disseminated intracellular infection, and a parainflammatory dermatosis. The pathogenesis of Sweet syndrome, a neutrophilic dermatosis, remains incompletely elucidated, but it is widely recognized as a hypersensitivity reaction to various stimuli, including malignancies, drugs, and infections.^[5]^ In our patient, the temporal association and the confirmation of mycobacterial elements in the skin strongly support the diagnosis of Sweet syndrome secondary to disseminated NTM infection.

The diagnostic journey, however, pointed to a deeper immunological abnormality. The occurrence of disseminated NTM disease in an apparently immunocompetent, HIV-negative adult is a hallmark of impaired IFN-γ-mediated immunity.^[6]^ Our patient was subsequently found to have high-titer AIGA, which is the defining feature of a distinct entity known as adult-onset immunodeficiency syndrome (AOID).^[7]^ This syndrome exhibits a striking predilection for elderly populations of Southeast Asian descent, a demographic predisposition potentially linked to specific HLA alleles such as DRB1 and DQB1.^[8,9]^ AIGA effectively neutralizes IFN-γ, a critical cytokine for macrophage activation and the control of intracellular pathogens, thereby predisposing individuals to a spectrum of opportunistic infections, with disseminated NTM being the most common.^[10]^

The co-presentation of AIGA-mediated AOID and Sweet syndrome in this context may be explained by a 2-stage hypothesis. The primary defect is the AIGA-induced blockade of the IFN-γ pathway, creating a permissive environment for the dissemination of M. massiliense. The subsequent robust, albeit dysregulated, neutrophilic host response to the persistent mycobacterial antigens is then manifested clinically as Sweet syndrome.^[11,12]^ This proposed model would carry significant therapeutic implications if validated. Management must extend beyond conventional antimicrobial therapy to address the underlying immune dysfunctio^n^.^[13,14]^ Our therapeutic decisions were guided by this framework. Rituximab (375 mg/m^2^ weekly for 2 doses) was chosen to deplete autoreactive B cells and curtail the production of pathogenic autoantibodies, an approach that has been reported to restore IFN-γ signaling in similar patients.^[13]^ The triple-antibiotic regimen (clarithromycin, moxifloxacin, linezolid) was designed to target rapidly growing mycobacteria, including M. massiliense, based on consensus guidelines,^[15]^ given the lack of susceptibility data. Corticosteroids were administered to rapidly control the severe neutrophilic inflammation of Sweet syndrome while antimicrobial therapy took effect. The observed rapid and satisfactory clinical response to this combined regimen robustly supports this comprehensive therapeutic strategy.

This case has limitations. The follow-up duration was limited to 3 months because the patient relocated and could not return. Consequently, long-term relapse risk and the durability of the response remain unknown. Based on the natural history of AIGA-AOID, periodic surveillance including clinical assessment, inflammatory markers, CD19 + B-cell counts, and imaging is recommended to detect recurrence early and guide the need for repeated rituximab dosing.

In conclusion, this case underscores a critical clinical pearl: the emergence of Sweet syndrome in an adult, particularly in an endemic region, should prompt an investigation for an underlying trigger. When discovered, disseminated NTM infection should serve as a red flag for an underlying immunodeficiency, specifically AIGA-mediated AOID. A high index of suspicion for this association is essential for timely diagnosis and the institution of dual-pathogen-directed and immunomodulatory therapy, which is crucial for achieving a successful outcome.

## Acknowledgments

The authors have no funding and conflicts of interest to disclose.

## Author contributions

**Conceptualization:** Jiajia Chen, Jiguang Guo.

**Investigation:** Jiajia Chen, Jiguang Guo.

**Supervision:** Jiajia Chen, Jiguang Guo.

**Writing – original draft:** Jiajia Chen, Jiajun Liu, Lili Wang.

**Formal analysis:** Zihao Han, Jiajun Liu.

**Methodology:** Zihao Han, Lili Wang.

**Resources:** Zihao Han, Lili Wang.

**Visualization:** Zihao Han, Jiajun Liu.

**Software:** Jiajun Liu.

**Writing – review & editing:** Jiguang Guo.
